# Ultra-Low-Power Sensor Nodes for Real-Time Synchronous and High-Accuracy Timing Wireless Data Acquisition

**DOI:** 10.3390/s24154871

**Published:** 2024-07-26

**Authors:** Tadeusz Sondej, Mariusz Bednarczyk

**Affiliations:** Faculty of Electronics, Military University of Technology, 00-908 Warsaw, Poland; mariusz.bednarczyk@wat.edu.pl

**Keywords:** data acquisition, data gathering, energy-efficient devices, real-time systems, synchronous data acquisition, ultra-low power, wireless sensor networks, wireless body sensor networks

## Abstract

This paper presents an energy-efficient and high-accuracy sampling synchronization approach for real-time synchronous data acquisition in wireless sensor networks (saWSNs). A proprietary protocol based on time-division multiple access (TDMA) and deep energy-efficient coding in sensor firmware is proposed. A real saWSN model based on 2.4 GHz nRF52832 system-on-chip (SoC) sensors was designed and experimentally tested. The obtained results confirmed significant improvements in data synchronization accuracy (even by several times) and power consumption (even by a hundred times) compared to other recently reported studies. The results demonstrated a sampling synchronization accuracy of 0.8 μs and ultra-low power consumption of 15 μW per 1 kb/s throughput for data. The protocol was well designed, stable, and importantly, lightweight. The complexity and computational performance of the proposed scheme were small. The CPU load for the proposed solution was <2% for a sampling event handler below 200 Hz. Furthermore, the transmission reliability was high with a packet error rate (PER) not exceeding 0.18% for TXPWR ≥ −4 dBm and 0.03% for TXPWR ≥ 3 dBm. The efficiency of the proposed protocol was compared with other solutions presented in the manuscript. While the number of new proposals is large, the technical advantage of our solution is significant.

## 1. Introduction

Synchronous data acquisition is required in many areas, including industry, science, sports, and medicine. It is often required in local or distributed measurement systems where communication can be wired, wireless, or mixed. In the case of wired communication, synchronization is easy to ensure. This is because of the possibility of transmitting a clock signal or predictable data latency in wired interfaces. Synchronization pulses can also be sent between the measuring system devices. In the case of wireless interfaces such as radio communication in Wireless Sensor Networks (WSNs), ensuring synchronization between devices is significantly difficult, mainly because of the existence of multiple clock sources. Various methods for clock synchronization in WSNs are well-known [1,2,3,4,5]. However, clock synchronization does not always guarantee the synchronization of sensor data obtained from many nodes, and the synchronization achieved by these methods can be insufficient [6,7]. This problem increases with some specific applications, which include data acquisition at high sampling rates, long-duration continuous sampling, nonlinear clock drifts, or when a low power consumption is required.

Synchronous data acquisition is crucial, for example, for structural health monitoring [7,8,9,10,11,12] and many applications of Wireless Body Area Networks (WBANs) [13,14,15,16]. Significant challenges in WBANs are described in [17,18,19,20] and include requirements for low-power technology, small sensor dimensions, multi-device synchronous measurements, and the recording of physiological parameters, data fusion, and dissemination. Widely used radio technologies such as Bluetooth Low Energy, ZigBee, ANT, and Ultra-Wideband can be used for internal communication in WBAN star or mesh topology. Unfortunately, these technologies do not always provide the synchronization required for data acquisition. This problem especially increases in the case of a star topology with many sensor nodes. Examples of such situations are synchronous data acquisition from multi-site sensors for monitoring physical human activity [21,22,23], multichannel electromyography (EMG) [24,25], synchronous electrocardiographic (ECG) and photoplethysmographic (PPG), multi-site PPG signals’ measurement for continuous pulse wave velocity [26], and cuff-less blood pressure monitoring [27,28,29]. Furthermore, in these applications, the recommended sampling frequency (*f_S_*) is at least 1 kHz [27,30], which is mostly hard to reach. In addition to synchronous data acquisition, a low energy consumption is highly desirable, particularly for battery-powered sensor nodes used in wearable medical electronics and IoT devices [31,32]. In addition, a low power design is most important for battery-free and energy-aware autonomous wireless sensor nodes [33,34,35,36,37].

Several sampling synchronization methods are available for multi-node wireless sensor networks. However, these proposals are limited in various aspects, such as the precision of time synchronization, data latency, power consumption, and the cost of implementation. In [12], GPS receivers were used for sampling synchronization in the multichannel wireless acquisition of seismic data, but they provided an expensive, high-power-consuming, large, and inconvenient solution. GPS-based synchronization in WSNs for vibration measurement was also presented in [38]. In this work, the synchronized monitoring of acceleration data was implemented. Unfortunately, the proposed sensor node was quite complex, with large dimensions and limited applications. Similarly, a GPS module was used in [39] to synchronize the sampling process of accelerometers. Unfortunately, there are no data on synchronization errors and power consumption. In [22], a wireless wearable multi-sensor based on ZigBee communication was presented. The proposed system consisted of three sensor nodes that measured signals with a sampling rate of only 30 Hz and data synchronization with an accuracy of only 24 ms. The power consumption depends on the packet size, and it is always over several milliamperes (mA). The use of the Bluetooth interface for synchronization in a multi-nodes WBAN was presented in [40]. A synchronization accuracy of 30 μs was obtained; however, it was necessary to modify the Bluetooth protocol stack. Moreover, the classic Bluetooth standard was used, and the power required for one sensor node was 110 mW (for *f_S_* = 500 Hz). In [16], Bluetooth Low Energy (BLE) and a custom protocol for simultaneously gathering multiple bio-signals from multiple body areas was used. The best results were achieved for data synchronization with a frequency of 40 Hz. This solution also required a modification of the BLE stack. Moreover, work [16] did not provide results for energy consumption and possible data throughputs. Multi-modal data acquisition system with BLE for human motion analysis was presented in [41]. Inertial measurement unit modules were connected via BLE to an Android smartphone. However, the frequency sampling was only 60 Hz and the synchronization accuracy was evaluated by simultaneously recorded video data. A similar solution for an accurate gait analysis during running was described in [42]. Data synchronization from many sensors was performed by software based on the time stamps transmitted from sensors. The synchronization error was estimated at approximately 2 ms, but there was no information about the sensor’s energy consumption. Wi-Fi-based sensor nodes for human gait monitoring were presented in [43]. For the synchronization of independent data acquisition systems, two methods were proposed: (1) a solution involving the repeated synchronization of the entire system by issuing network requests to extract sensors’ data with a very low latency, and (2) a method where a synchronizing message is sent only at the beginning of a data-logging session. The developed algorithm allows data acquisition from six pressure sensors and two IMUs, ensuring a 24.6 Hz sampling rate and a 1 ms synchronization accuracy. Unfortunately, there was a lack of information about the energy consumption of the sensors. Another solution based on Wi-Fi sensor nodes was presented in [44]. Although a high *f_S_* (≥1 kHz) was achieved, there was no information about how the data was synchronized. Additionally, the current consumption was as high as 229 mA (unfortunately, the supply voltage was not specified). In [45], a wireless, Wi-Fi-based, group-synchronized neural recording system, which supported real-time multi-subject brain–computer interface and event-related potential, was presented. An additional wireless trigger was used to synchronize the data. A synchronization error of 237 μs was obtained. Unfortunately, there was no information about the energy consumption of the node. A low-cost Wi-Fi module was also used in a wireless data acquisition system based on MEMS accelerometers for the operational modal analysis of bridges [46]. In such systems, a significant challenge is the synchronization of data captured by multiple sensor devices. In [46], it was assumed that the synchronization error would be less than 1/10 of the sampling period. A distinct timer maintained by the network access point (the router) was used to synchronize many wireless sensors. A synchronization error of approximately 2 ms was obtained. Unfortunately, the average current consumption for one sensor during transmission was as much as 125 mA (for 12 V battery). A timing slot algorithm for a multichannel sub-1 GHz low-power wide-area network (LPWAN) was presented in [47]. After applying real-time clock compensation, a 5 µs synchronization error was obtained during ta continuous acquisition of 10 s at *f_S_* = 10.24 kHz. However, the data from each sensor (one channel) were transmitted at five different radio frequencies. Unfortunately, the energy consumption was not reported in this study. In addition, there are known custom-made solutions that do not use popular radio interfaces. A protocol based on time-division multiple access (TDMA) for bidirectional sensor-based station communication was presented in [48]. A variance in synchronization error of 7.6 μs was obtained with a clock base of 32.768 kHz. The power consumption was not reported in this study. Another energy-efficient solution based on TDMA for WBAN communication was presented in [49]. The industrial, scientific, and medical (ISM) 433 MHz band was used to implement the low-duty cycle MAC protocol. An average power of 2.4 mW was obtained at 1250 bit/s sampled data, but synchronization error was not reported.

The problems of data synchronization and energy saving have been also addressed in numerous other studies. However, many of them are limited only to the modeling and simulation of the proposal, for example [20,31,50,51]. There have only been a few implementations under real conditions, where there were many more factors affecting the final results. Many studies have limited their analysis to wireless and energy-efficient sensors, but a star topology has not been considered [33,52]. There are also known solutions implemented in application-specific integrated circuit (ASIC) devices [53,54].

The aforementioned studies indicate that the problem of data sampling synchronization in wireless sensor networks is still under consideration. Another significant problem is the acquisition of data in a synchronized manner with a very low energy consumption. As indicated in [55], this approach is gaining increasing attention owing to the growing interest in IoT battery-powered devices, energy-critical applications, and especially in WBAN applications.

From the literature survey, it was concluded that there are many TDMA MAC protocols in WBANs for data transmission. All protocols have many issues, such as energy consumption and packet collision, as it is the main concern in WBANs, which is being resolved in this study with the concept of data synchronization.

In this paper, a proprietary protocol for data synchronization, together with synchronized access to media, is proposed. The method is TDMA-oriented, not only in the sense that every node has its own reserved time slot for data transmission. but also in the sense that each node starts the sampling cycle at the same time. The idea behind the proposal is that each node in the first time slot of the TDMA cycle can precisely determine the start time of the sampling events. In addition, the proposed method operates with a low sensor energy consumption and implementation costs. For a given TDMA cycle, the duration of the time slots decreases as the number of nodes increases, and vice versa.

Aside from the proposed proprietary protocol, in this work, a custom-based, ultra-low-power, wireless multi-nodes sensor network for synchronous data acquisition was designed, prototyped, and experimentally evaluated. The proposed solution can be used for the simultaneous multi-site measurement of many human physiological, motion, and pressure signals [16,26,41,43] or other healthcare/medical devices.

The main contributions in our work are high-accuracy data acquisition synchronization; ultra-low energy consumption; high-throughput real-time data with a constant latency of data acquisition; a very low computational complexity; and low implementation costs (because our solution is based on SoC platforms and implements a light-weight TDMA method without the need to exchange various messages).

The rest of this paper is organized as follows. In Section 2, the proposed system architecture, media access control (MAC) protocol, and sensor node design are described. Section 3 describes the implementation of the proposed method. The experimental results are presented in Section 4. In the next section, the proposed solution is evaluated and discussed (Section 5). Finally, the conclusions of this study are presented in Section 6.

## 2. System Design

### 2.1. Topology of Data Acquisition System

**Figure 1:**
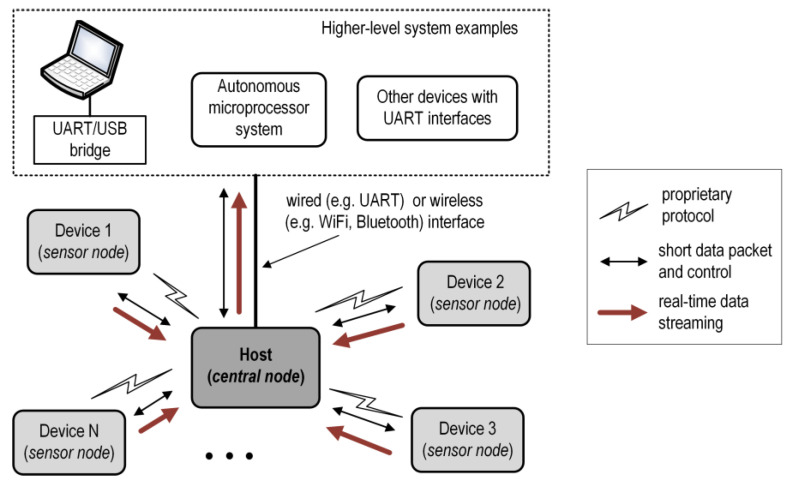
Block diagram of multi-nodes system for wireless synchronous data acquisition.

The structure of the multi-node wireless system with synchronous data acquisition is shown in Figure 1. 

Sensor nodes (Device 1…N) are responsible for the sampling of signals (like EMG, ECG, accelerometers, etc.) and transmitting them as raw data to the central node (Host) in real time (data streaming). Then, the Host transmits the collected data to a monitoring station at a higher level through a wired (e.g., UART) or wireless (e.g., Wi-Fi and BLE) interface. These Devices cannot communicate with each other. We assume that the distance between the Devices and the Host is small (approximately a few meters, as in the WBAN network). This distance could be greater, but this would be at the cost of worsening the synchronization parameters and increasing the energy consumption. Therefore, this solution is not considered. Both the Host and Devices can be implemented in a specialized SoC technology with all the necessary electronic circuits and parts for a given system, including a transceiver and a microcontroller. In some cases, the Host can also be equipped with an additional application processor (e.g., nRF5340 dual-core SoC [56]) for data processing and/or other tasks and can be powered from high-capacity sources (e.g., USB). In our architecture, the Host acts not only as a central node to collect data, but also as a master to control channel access.

To avoid data collisions during transmission and energy consumption owing to retransmission, the TDMA technique is used for media access control. The time slot assignment in the TDMA scheme is made a priori, and the Host is responsible for it, as well as for the sampling synchronization within Devices. The principle of time slot allocation is very simple. The first slot always belongs to the Host, and the others to the Devices. In addition, each element of the system has its own clock source for determining the interval of the sampling events. Additionally, to achieve a low energy consumption by Devices and synchronized data acquisition during sampling events, some modifications can be made, such as SoC peripheral hardware event control, a Direct Memory Access (DMA) controller, SoC firmware optimization, and sleep SoC during non-transmission periods.

### 2.2. Media Access Control for Proposed Protocol

**Figure 2:**
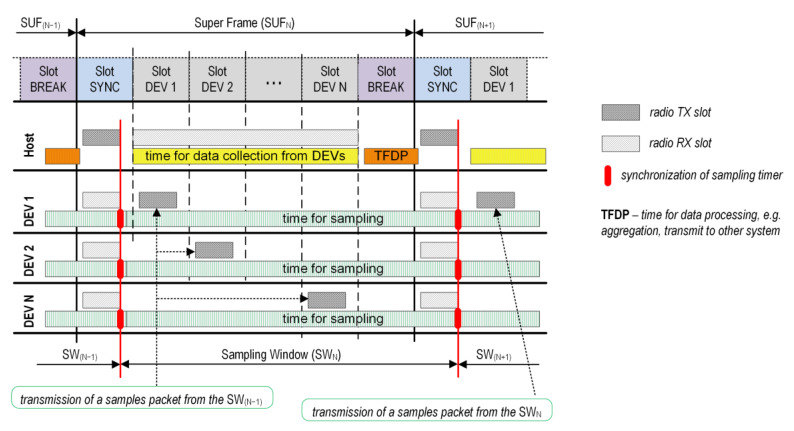
Super Frame structure for proposed solution.

Communication between the elements of the system is based on the TDMA scheme with specific control strategies to realize a low energy consumption, real-time signal sampling, high-accuracy data synchronization, and a Device-to-Host bulk data streaming direction. The proposed basic timing slot configuration is shown in Figure 2.

The TDMA scheme is based on a Super Frame (SUF) divided into three types of slots: SYNC, DEV, and BREAK. The first slot (SYNC) of each Super Frame is used by the Host to transmit the synchronization packet . All the sensor nodes start listening to the sync packet, shortly before they expect it. Owing to the broadcasting nature of the transmitted packet, it can be received by all participants in the system. The end of the received sync packet determines the moment of the sampling timer synchronization and the beginning of the Sampling Windows (SW) in the receiving nodes (red line in Figure 2). The sensor nodes then start to sample the measured signals with an interval that can be individually defined for each node. The assignment of DEV slots to the sensor nodes establishes a TDMA schedule and guarantees deterministic packet transmission. Each sensor node receives one time slot (DEV 1…DEV N), during which, it can transmit data to the Host. In the SUF_N_ frame, the sensor node can transmit data from a previous sampling window (SW_N_). The BREAK slot is used by the Host to process the data collected from the sensors.

**Figure 3:**
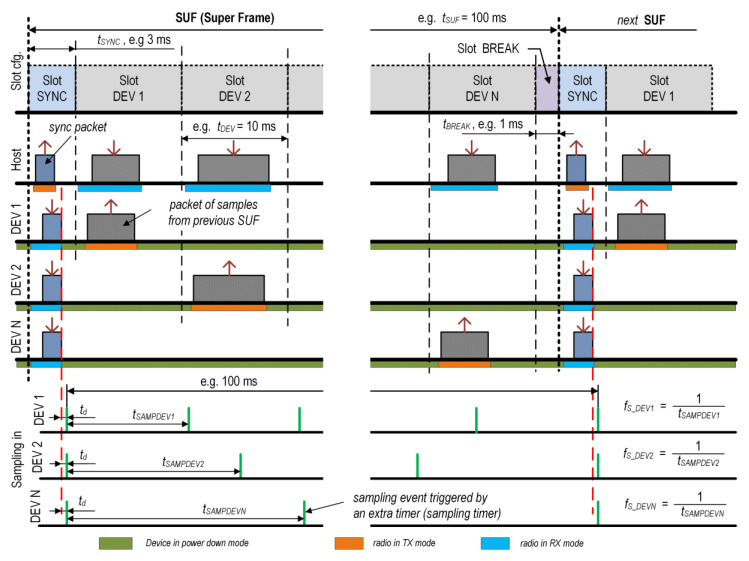
Frame and timing slot configuration for proposed MAC protocol.

A detailed configuration of the time slots for the proposed protocol is shown in Figure 3. The total duration of the Super Frame is *t_SUF_*, which is equal to, e.g., 50 ms, 100 ms, and 200 ms. This depends on the number of nodes, types of measured signals, and target application. The SYNC slot always has a constant duration (e.g., 3 ms). This value is sufficient to transfer even several hundred bytes from the Host to the Devices, however, in our proposal, it is used to send only a few bytes of the synchronization packet.

If the sync packet is received (red dashed line in Figure 3), the receiving nodes synchronize with the local extra sampling timer. Time *t_d_* is required for this action. This synchronization restarts with the sampling timer. In the next step, the sensor nodes will start to transmit data in their assigned slots. The duration of each slot depends on the number of sensors, but the sum of all the DEV, SYNC, and BREAK slots cannot exceed *t_SUF_*. This is configured during the setup process prior to the system initialization. In a TDMA scheme, a guard interval of 50 µs between time slots is used to reduce interference and prevent overlapping transmissions due to clock drifts at the sensor nodes. If the Device is not in the RX (SYNC slot) or TX (DEV slot) mode, it spends time in a power-down mode to minimize energy consumption. A short-term wake-up may only occur during the sampling process, unless it is not implemented in the hardware approach (without the participation of the SoC CPUs). An extra BREAK slot with a constant duration (e.g., 1 ms) is reserved for data processing from all sensor nodes, for example, for DMA transfer initialization (e.g., from the Host to a higher-level system). This slot is assigned only to the Host.

### 2.3. Data Synchronization

To achieve real-time signal sampling and high-accuracy data synchronization, we propose a hardware/software method that determines the appropriate starting moment for signal sampling and the packetized data sent in dedicated time slots. To do this, we use the fixed length of the synchronization packet sent in the SYNC slot. The end of the frame while receiving it indicates the moment of synchronization with the Host. However, to maintain the precise sampling of the sensors (Devices), an additional hardware sampling timer is started immediately upon the reception of a sync packet. This counter is already preconfigured by the software, so that its first interruption (first sampling event in Figure 3) occurs after a fixed time of *t_d_*. Delay *t_d_* depends primarily on the speed of the CPU used in the SoC and on how to trigger the start of the sampling timer. This start can be performed by software or hardware using an appropriate connection controller between the peripherals in the SoC. The slight *t_d_* dispersion may be due to the radio propagation time of the sync packet, but it is insignificant. The sampling frequency can be the same (*f_S_DEV_*_1_ *= f_S_DEV2_ = f_S_DEVN_*) or different for each of these Devices. The most important step is to maintain the first sampling event at the same time. It is also assumed that the sampling period in each Device is uniform during the SUF frame. Therefore, *t_SUF_* should be an integer multiple of *t_SAMPDEVN_*.

## 3. Implementation of the Proposed Solution

### 3.1. Test Bench

**Figure 4:**
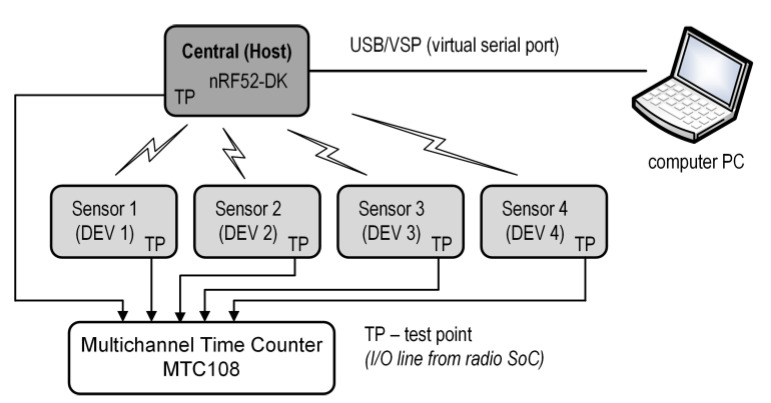
System configuration for tests of the proposed solution.

To implement and experimentally test the proposed solution, we developed a system consisting of four identical sensor nodes (Sensor) and a central unit (Central). Figure 4 shows the diagram of a test bench, including the Sensors, Central, PC computer, and multichannel time counter. 

The Sensors and Central have an output line (TP—test point) directly from the used SoC, which are wire-connected to the multichannel precise time and frequency counter MTC108 [57]. MTC108 is used to measure the frequency of the Central and Sensor clocks, and to measure the time dependencies of the proposed protocol. Communication between the Central and the PC computer is carried out in the virtual serial port mode. This makes it very easy to display various messages and data in real time on a computer, for example, in the free SerialPlot software (Version: 0.12.0). The Central is implemented on the nRF52-DK development board. The nRF52-DK board contains a specialized nRF52832 (nRF52) SoC by Nordic Semiconductor [58], low-frequency quartz (changed from the original 32.768 kHz to 40.000 kHz), and a J-Link debugger with a built-in virtual serial port.

### 3.2. Sensor Node Design

**Figure 5:**
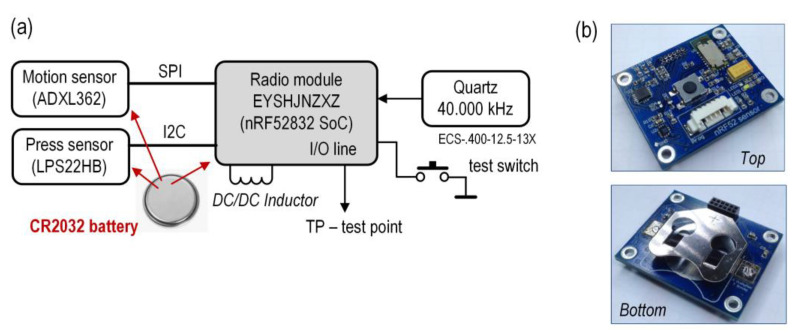
Block diagram (**a**) and the photo (**b**) of the designed sensor board.

A custom-designed board was used to make the Sensors. Several boards of the same construction type were constructed. A block diagram and photograph of the prototype board are shown in Figure 5.

The main element of the sensor board is an EYSHJNZXZ radio module by Taiyo Yuden (Taiyo Yuden Co., Ltd., Tokio, Japan) [59] with an embedded 32 MHz source clock and a PCB pattern antenna. It is a very small (11.3 mm × 5.1 mm) module with an nRF52 SoC (the same as that in the Central). Although this module is no longer produced, our proposed solution can use many other modules with nRF52 or similar SoCs. The nRF52 SoC includes an energy-efficient high-performance ARM-Cortex-M4F core, running at 64 MHz, many peripherals and interfaces, Flash and SRAM memories, and a 2.4 GHz multi-protocol radio controller. An important feature of nRF52 is the Programmable Peripheral Interconnect (PPI) that enables peripherals to interact autonomously with each other using tasks and events independent of the CPU. The use of the PPI technique and a DMA controller significantly reduces the power consumption. An external low-frequency quartz and a coil for the built-in DC/DC regulator are connected to the radio module. As for the Central, to achieve, for example, the 100 ms TDMA frame duration, we use a 40.000 kHz low-frequency clock. An ultra-low-power ADXL362 (Analog Devices Inc., Wilmington, DE, USA) accelerometer and an LPS22HB (STMicroelectronics, Geneva, Switzerland) pressure sensor are used as exemplary sensors. One I/O line (TP) of nRF52 is used in the experiments. The designed sensor board is powered directly using a CR2032 battery. The software (nRF52 SoC firmware) implementing the proposed protocol is written in C, in the Keil MDK development environment, with the support of the Nordic SDK libraries.

**Figure 6:**
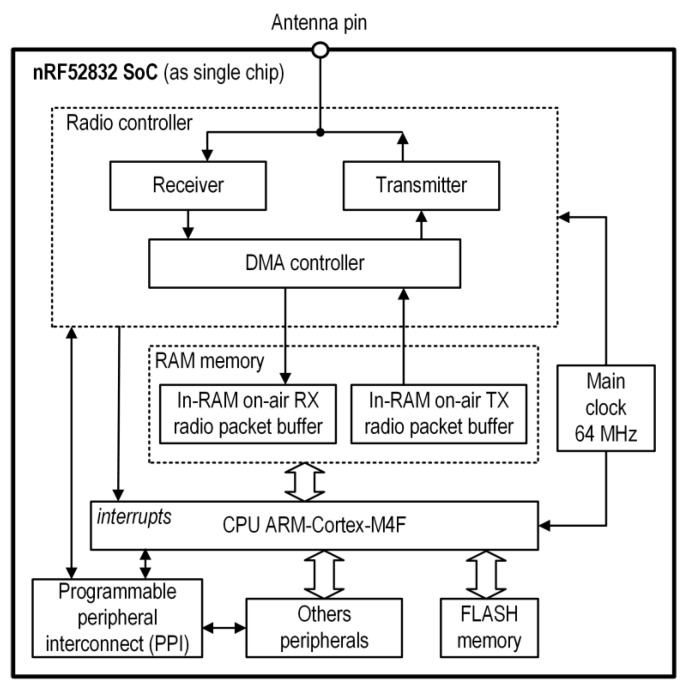
Simplified block diagram of the nRF52832 SoC.

In the nRF52 SoC used in the Sensor, one of the main and most important elements for our solution is the radio controller and the way it communicates with the microcontroller. Figure 6 shows a simplified block diagram of the nRF52 SoC with the important elements of the radio controller (RC). The RC is one of the peripherals of the SoC. It has the following features: a [2400–2500] MHz or [2360–2460] MHz frequency band with a 1 MHz channel spacing; supports Frequency-Shift Keying (FSK) modulation; a data rate of 1 Mb/s or 2 Mb/s; a transmitter output power from −40 dBm to +4 dBm; and a receiver sensitivity from −89 dBm to −96 dBm, depending on the radio configuration. The RC uses a fixed channel with a center frequency of 2421 MHz. This channel is selected to avoid interference with channels 37–39 used in Bluetooth Low Energy and to mitigate the influence of IEEE 802.11 networks (Wi-Fi). This frequency value is located at the edge of Wi-Fi channel 1. For operations using a 20 MHz channel spacing in Wi-Fi networks, the transmitted spectrum should have a −20 dBr (dB relative to the maximum spectral density of the signal) at an 11 MHz frequency offset relative to the center frequency (according to IEEE Std 802.11™). Consequently, the signals received from Wi-Fi networks are negligibly small.

As mentioned earlier, the manner in which the RC interacts with the microcontroller is very important. The RC has its own DMA controller, which provides direct access to the RAM of the microcontroller. In the RAM, there are dedicated buffers in which the received or transmitted on-air data frame is saved. Therefore, during the transmission or reception of data by the RC, the microcontroller can be in sleep mode. This significantly affects energy savings. In addition, various events occurring in the RC, such the packets sent or received, interrupt the microcontroller directly. Moreover, in our design, we use PPI to control the other peripherals via the radio, without the need to wake up the microcontroller from sleep mode. For example, a PPI task involves a radio disabled event with the main clock stop task.

**Figure 7:**
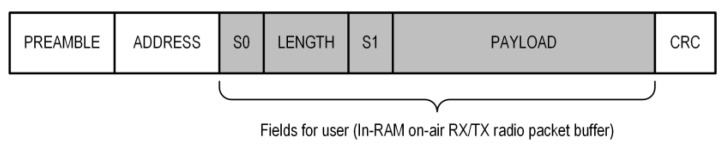
On-air radio packet layout of the nRF52 SoC.

The Radio packet buffer (for the RX or TX, as shown in Figure 6) is an element of the basic on-air radio packet of the nRF52 SoC. The layout of the packet is shown in Figure 7.

The PREAMBLE and ADDRESS fields are set once during the configuration of the radio controller, and the S0, LENGTH, S1, and PAYLOAD fields (as user fields) can be set before each frame is sent, whereas the CRC field is calculated and set by the hardware. The total number of bytes of fields for the user cannot exceed 258.

### 3.3. Configuration of the Proposed Protocol

**Figure 8:**
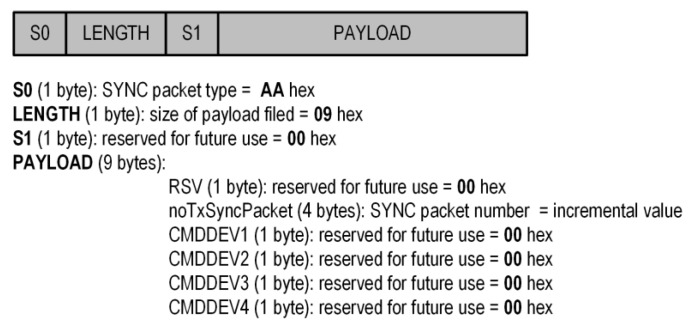
Contents of the sync packet for implementation proposed protocol.

The proposed protocol was implemented using a previously described Sensor and Central. The SUF duration (*t_SUF_*) was assumed to be 100 ms, the SYNC slot duration (*t_SYNC_*) was assumed to be 3 ms, and the BREAK slot duration (*t_BREAK_*) was assumed to be 1 ms. The number of sensor nodes in the system was four. Therefore, the slot duration (*t_DEV_*) for one sensor node (DEV) was 24 ms. The length of the sync packet was fixed at 12 bytes. The contents of the sync packet are shown in Figure 8.

For the implementation of the proposed protocol, the first four bytes have a constant value and allow the Sensor to check whether the received packet is a sync packet. The noTxSyncPacket field is incremented when this packet is sent and is used to investigate the number of lost packets. On the other hand, the CMDDEV1…4 fields in our case have a value of 0, but may be used in the future to send any data from the Central to the Sensors. However, as we mentioned earlier (Section 2.2), for our solution, the content of the sync packet is not important, but the moment of its receipt is.

**Figure 9:**
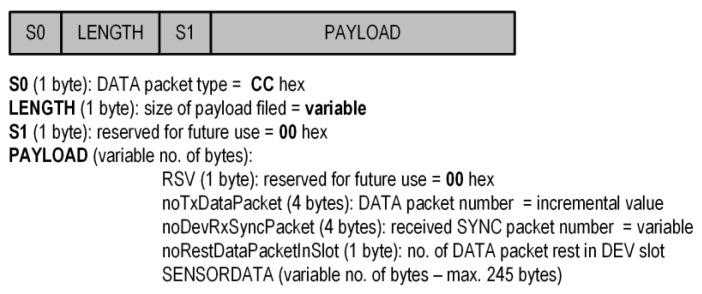
Contents of the data packet for implementation proposed protocol.

The data packets (Figure 9) sent from the Sensors to the Central are of variable lengths.

The S0, S1, and RSV fields have a fixed value, whereas the others are variable. The noTxDataPacket field is incremented when this packet is sent, and the noDevRxSyncPacket field contains the number of the received sync packets. These fields allow for the investigation of the number of lost packets and the management of data on the Central side. The noRestDataPacketInSlot field contains the number of data packets to be sent in the assigned DEV slot during the SUF frame. This situation occurs when the amount of data to be sent from the Sensor to the Central does not fit into one on-air frame. The SENSORDATA field has a variable length and contains the data sent to the Central. In our case, these are samples of accelerometer (ADXL362) signals measured by the Sensor.

### 3.4. Samples Acquisition in the Sensor

In the implementation of the proposed solution, in the Sensor, samples of measurement signals were acquired in each sampling event (see Figure 3) in two ways: (1) by simulating the sample values and (2) by reading samples from a real sensor (ADXL362) connected to the nRF52 SoC microcontroller (see Figure 5a). The sampling events were determined using an additional nRF52 SoC low-power timer (RTC2 instance), referred to in our proposal as the sampling timer. The simulation consisted of entering the data directly into the SENSORDATA field of the data packet (see Figure 9). In turn, data from the three-axis ADXL362 accelerometer were read in real time, every 10 ms duration, in each sampling event using the SPI interface with an 8 MHz clock. With this clock, reading one sample of data from the three axes took approximately 25 µs. Therefore, in each sampling event of the SUF_N_ frame, every 10 ms, the microcontroller was woken up (by the sampling timer). Then for approximately 25 µs, the data from the accelerometer were read and saved to the buffer, and the microcontroller was put back to sleep. Subsequently, in the next frame (SUF_N+1_), the data collected in the buffer were transmitted to the Central.

### 3.5. Power Measurement

Because one of the goals of our study was to minimize the energy consumption of the Sensor, it was necessary to precisely measure the power, especially under dynamic conditions. Energy consumption depends on the hardware solutions and microcontroller software. Therefore, we used the ULINKplus adapter for sensor node implementation and testing. ULINKplus is an innovative and useful adapter for simultaneous debugging and precise energy measurements. The ULINKplus adapter stands out from the dynamic power measurement devices currently available on the market. It has a very high sampling rate (up to 20 MHz), wide measurement range, high resolution (16-bit A/C), and high accuracy, and it can correlate current and voltage measurements in the program code being executed. In addition, the ULINKplus adapter allows for the easy calculation of energy over a selected time interval (between two cursors). Owing to this, in the used nRF52 SoC, clocked at 64 MHz, it was possible to accurately synchronize the power and energy consumption with the executed program code.

**Figure 10:**
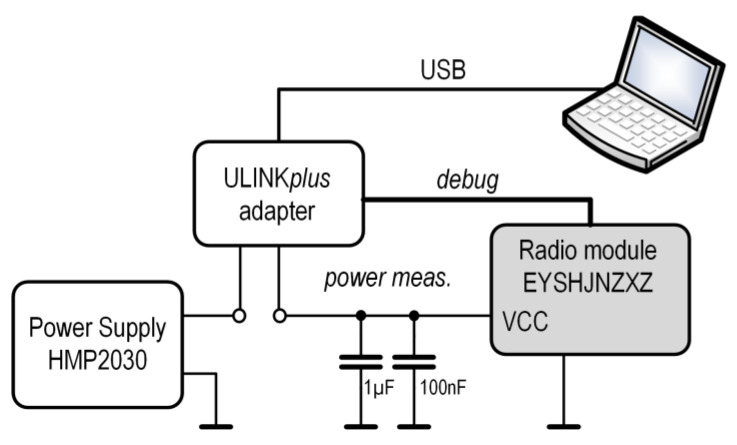
Block diagram of the power consumption measurement in radio module.

The power measurements for the Sensor radio module were performed according to the configuration shown in Figure 10.

An HMP2030 power supply was used as the energy source. The ULINKplus enabled precise measurements of the voltage, current, and energy. These measurements concerned only the radio module, as different types of sensors (e.g., accelerometer and biopotential analog front end) can be unique in terms of their overall energy consumption. Moreover, radio transceivers require most of the power required for typical wireless sensors. To accurately measure the impulse current changes, the decoupling capacitors of the radio module were only 1 µF and 100 nF. The selection of these capacitors is important, because their large capacity makes it impossible to observe impulse changes in the current, for example, when receiving or transmitting very short data packets or during hardware-software processes inside the nRF52 SoC.

## 4. Experimental Results

### 4.1. Base Frequency Measurements

In both the Central and Sensors, the base clock was the low-frequency clock (LFXO) of the nRF52 SoC. A crystal resonator (ECS-400-12.5-13X, ECS Inc., Lenexa, KS, USA) was used. The LFXO oscillator in the toggle-out mode (*f_LFXO_* divided by two) was connected to the TP line via the PPI. The CPU of nRF52 was in sleep mode. Frequency measurements were performed using an MTC108 (Frequency Mode, Gate = 10 s, and Samples Number = 10). The measurement results are shown in Table 1.

Two cases were tested. The first one was with the Central based on nRF52-DK, and the second was with the Central based on the Sensor board. The mean frequencies were almost the same, but the standard deviation (StdDev) was the highest for the Central based on the nRF52-DK. This was owing to the power supply of the nRF52-DK board from the USB of the desktop computer.

### 4.2. Super Frame Duration Measurement

The SUF durations in the Central and Sensors (generated from the received sync packets) are shown in Table 2. For these and subsequent measurements, MTC108 was in the multichannel Time Interval mode, and the samples number was 1000. Measurements were made for the Central and Sensors simultaneously. The results are the averages of three independent measurements.

For all Sensors, the StdDev of *t_SUF_* was approximately twice as large as that of the Central and was approximately five clock cycles of the nRF52 CPU (CPU clock = 64 MHz). The larger value of StdDev in the Sensors resulted from the additional dispersion of the detection of the moment of receiving the sync packet.

### 4.3. Super Frame Synchronization Measurement

**Figure 11:**
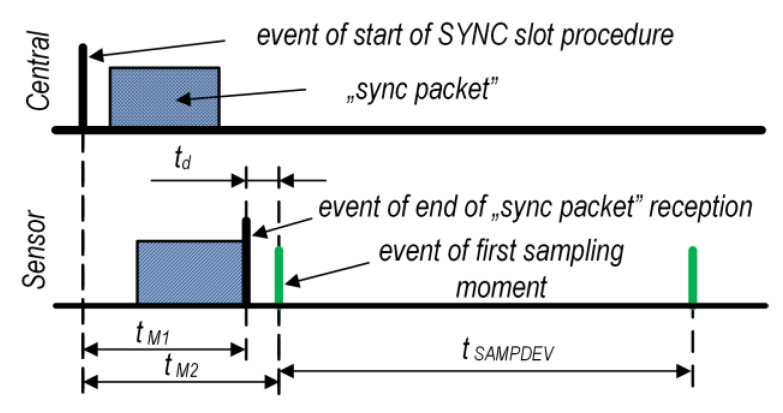
Timing definition for Central–Sensors synchronization measurements.

The next measurements concerned the achievement of the SUF synchronization moment between the Central and the Sensors. To obtain this, the time *t_M_*_1_ was introduced and measured, as shown in Figure 11.

The *t_M_*_1_ is the time between the start of a SYNC slot at the Central and the reception of the end of the sync packet at the Sensor, as shown in Figure 11. Measurements were made for two distances between the Central and the Sensor: 0.5 and 1.0 m. The results are shown in Table 3.

For the proposed protocol configuration, the measured *t_M_*_1_ is approximately 264 µs (with a sync packet length of 12 bytes), which is slightly higher for a distance of 1 m. The *t_M_*_1_ is highly dependent on the length of the sync packet. Therefore, the length of this packet must be constant for the adopted protocol implementation. The StdDev for all the sensors is very small and does not exceed 74 ns.

### 4.4. Sampling Moments Synchronization Measurement

For synchronous data acquisition, it is important to ensure the same sampling moments in all Sensors (as well as in the Central). For this, time *t_M_*_2_ was introduced and measured, as shown in Figure 11. The distance between the Central and the Sensors was 0.5 m. Table 4 presents the results.

It can be observed that the maximum difference in *t_M_*_2_ between the sensors was not greater than 1 µs. The higher StdDev value (compared with Table 3) was the effect of additional dispersion caused by the start of an extra timer for the sampling process.

Knowing the average times of *t_M_*_1_ and *t_M_*_2_, time *t_d_* can be calculated as the difference between the obtained averages. In our implementation, based on an nRF52832 SoC, we obtained *t_d_* = 47.318 µs. The average value of StdDev given in Table 3 and Table 4 is 66 ns, so it is approximately four CPU clock ticks.

### 4.5. Packet Error Rate Measurements

**Figure 12:**
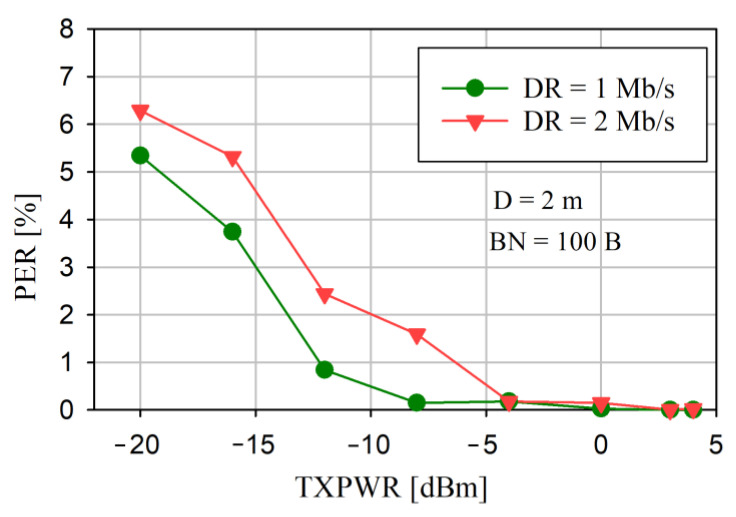
PER versus TXPWR for different data rates.

The Packet Error Rate (PER) is the ratio (in percent) of the number of test packets not successfully received by the Central to the total number of test packets sent to the Central (from the Sensor). Simulated test packets, one per slot, with known contents, were sent from the Sensors. Therefore, the Central could easily verify the correctness of the received packets. In the case of at least one byte of corruption, the entire packet was discarded. To estimate the PER, 10 thousand packets were sent for each test. The PER was measured by considering several factors: the Sensor’s transmitter output power (TXPWR), the data rate (DR) of the radio, the number of data bytes transferred (BN) (in the PAYLOAD field) sent in the packet, and the distance (D) between the Sensor and the Central. In the on-air transmitted data packet of the nRF52 SoC, in addition to the payload data, there were also additional fields, for example PREAMBLE, ADDRESS, and CRC (see Figure 7). However, in the performed tests, BN was the payload size only. The results of PER versus TXPWR for two DR and a constant BN = 100 bytes with D = 2 m are presented in Figure 12.

The PER did not exceed 0.18% for TXPWR ≥ −4 dBm. For TXPWR ≥ 3 dBm, the PER ≤ 0.03%. It is obvious that increasing the DR or decreasing the TXPWR increased the PER.

**Figure 13:**
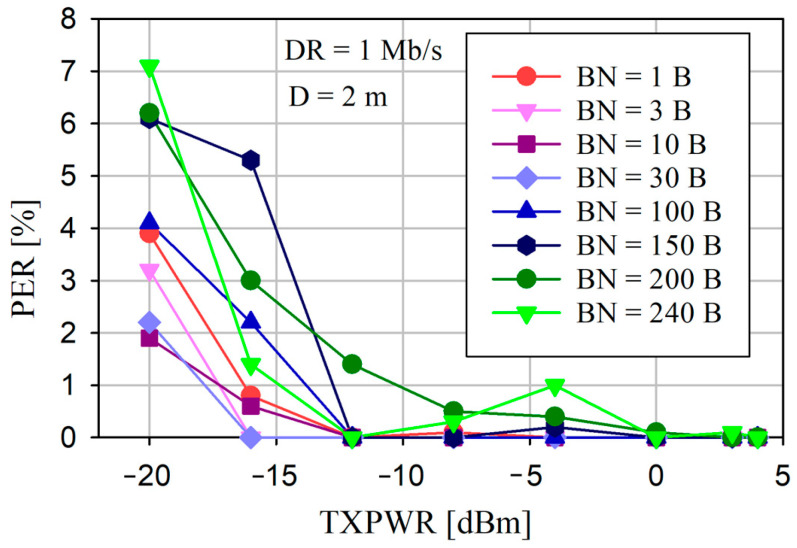
PER versus TXPWR for different BNs and constant DR (1 Mb/s) and D (2 m).

The PER versus TXPWR results for various BNs and a constant DR = 1 Mb/s and D = 2 m are shown in Figure 13.

Increasing BN increases the PER, however, it is highly dependent on the TXPWR. For TXPWR ≥ 0 dBm, the PER value does not significantly depend on BN and does not exceed the value of 0.1%. 

**Figure 14:**
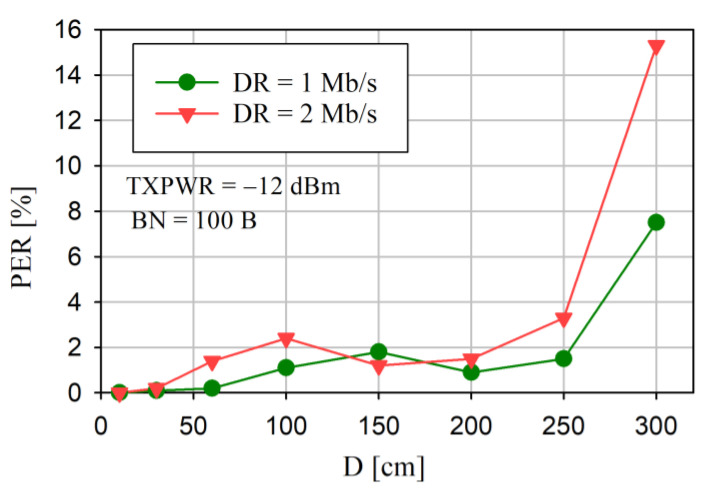
PER versus D for different DRs and constant TXPWR (−12 dBm) and BN (100 B).

The results of the PER versus D for various DRs, a constant TXPWR, and a BN = 100 B are shown in Figure 14.

Because even small PER values were not observed for TXPWR ≥ 0 dBm and D ≤ 3 m, the test was performed for TXPWR = −12 dBm. For the adopted measurement conditions, the PER did not exceed 2% for D ≤ 2 m. For D = 3 m, a large increase in the PER was observed, especially for DR = 2 Mb/s.

### 4.6. Sensor Radio Module Power Measurements

**Figure 15:**
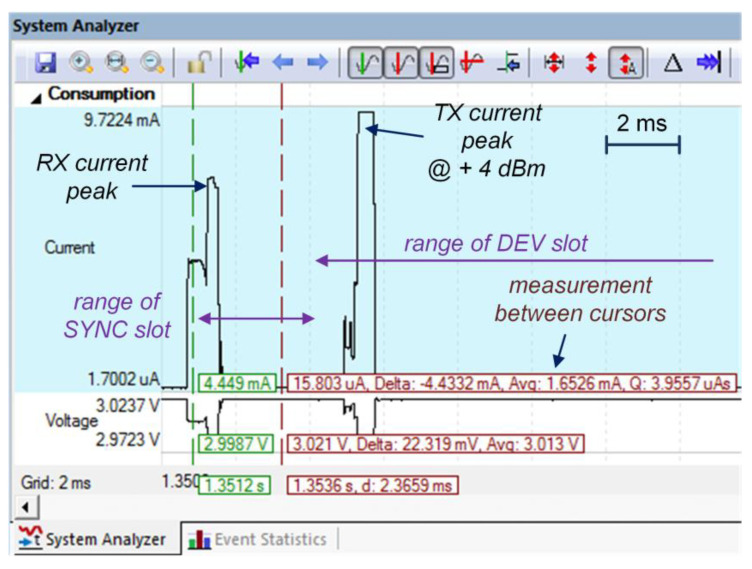
Example of Sensor radio module current consumption during RX and TX phases.

To measure the power consumption of the Sensor radio module, a ULINKplus adapter (ARM Keil, Aschheim, Germany) was used, as shown in Figure 10. This adapter cooperates with the Keil MDK environment, which supports the energy calculations. An example of the radio module power consumption (Current and Voltage) during the RX and TX phases is shown in Figure 15.

In this example, two momentary increases in the radio module current consumption can be observed. One peak was caused by the CPU wake-up and then turning on the transceiver RX mode. This was in the range of the SYNC slot. The other peak was caused by the CPU wake-up again and then preparing the data packet (100 bytes in size) and switching the transceiver to the TX mode at a power of +4 dBm.

**Figure 16:**
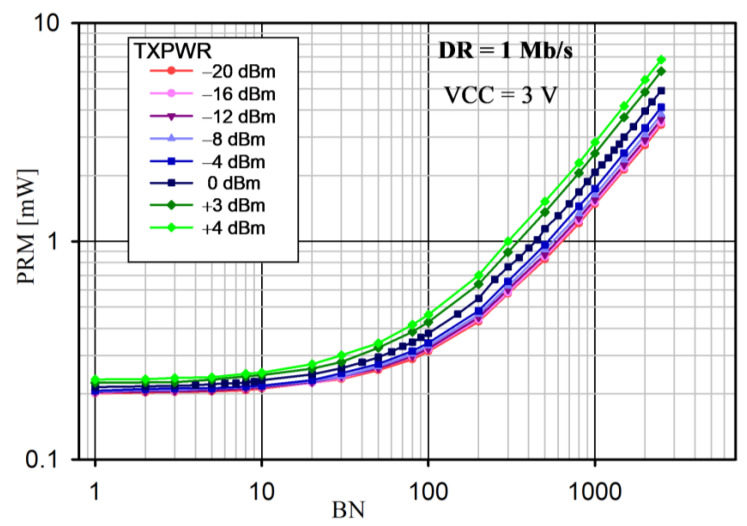
Radio module power consumption versus BN for different TXPWRs and DR = 1 Mb/s (VCC = 3.0 V).

**Figure 17:**
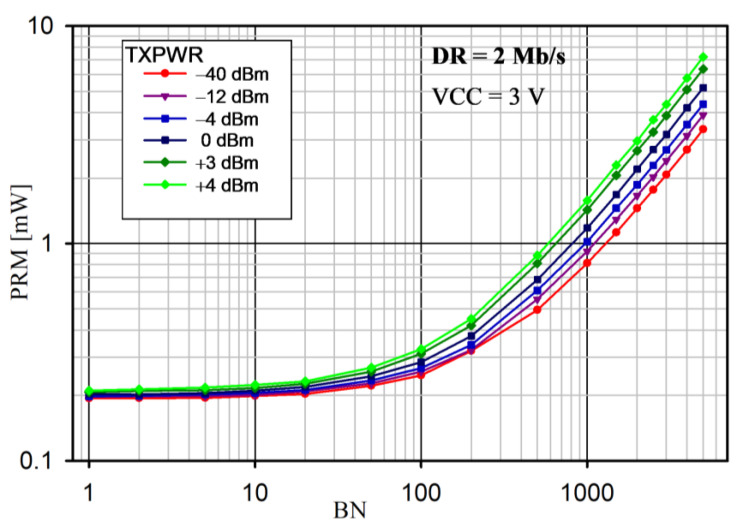
Radio module power consumption versus BN for different TXPWRs and DR = 2 Mb/s.

The radio module power consumption (PRM) versus BN for various TXPWRs for the proposed protocol is shown in Figure 16 and Figure 17 for DR = 1 Mb/s and 2 Mb/s, respectively. 

Note that, if the BN exceeded the allowable size of the PAYLOAD field for the on-air frame, another frame was sent. PRM was the mean value measured over 8 s. The radio module supply voltage was 3.0 V and the internal DC/DC converter was on. The slot duration was *t_DEV_* = 24 ms. For this reason, the maximum numbers of BNs were 2500 and 5000 for DR = 1 Mb/s and 2 Mb/s, respectively. In each case, the power consumption increased with an increase in the number of transmitted bytes (BNs) and transmitter power (TXPWR), which is obvious. This is because the highest power consumption occurred when the nRF52 CPU was on and when the transceiver was active (RX or TX mode). Moreover, if BN > 10, the power consumption significantly depended on the DR (it was higher for 1 Mb/s than for 2 Mb/s, because it took a longer time to transmit the data).

## 5. Evaluation and Discussion

### 5.1. Synchronization of Data Acquisition

One of the main goals of this study was to achieve synchronous data acquisition in distributed wireless sensor networks. It was assumed that the synchronization of the sample reading for all nodes would not be worse than 100 µs. For this purpose, a proprietary protocol based on the TDMA technique with the organization described in Section 2.2 was proposed. The following configuration parameters were adopted in the TDMA cycle: a *t_SUF_* duration of 100 ms and a device slot duration *t_DEV_* of 24 ms. Both the Central and the Sensors used a base clock equal to 40.000 kHz, which gave a period of exactly 25 µs. The experimental tests confirmed that the reproduced mean value of the *t_SUF_* duration for the Sensors (see Table 2) was 100.000 ms and the standard deviation was only approximately 80 ns. However, in saWSNs, it is important to achieve the same starting point for the signal sampling moments. In the proposed solution, the maximum difference (*r*) between the first sampling moments (Figure 11, *t_M_*_2_) of all the Sensors was 0.826 µs. It should be noted that the *r* difference could increase with the next sampling moments within the *t_SUF_* duration. This depended on the sampling rate and stability of the base clocks in the Sensors (see Section 5.5).

It is also worth paying attention to the value of the delay *t_d_* (see Figure 3 and Figure 11). The average *t_d_* in our implementation was approximately 47 µs. This was a fixed value and did not affect the accuracy of the sampling synchronization. From the perspective of the synchronization of data acquisition in the saWSN, the measure of the dispersion of synchronization moments (described as standard deviation) is more important. In our implementation, it was only 66 ns, which resulted in approximately four clock cycles. Achieving such a small dispersion was possible using the PPI controller, appropriate interrupt priorities, and optimizing the microcontroller program code.

**Figure 18:**
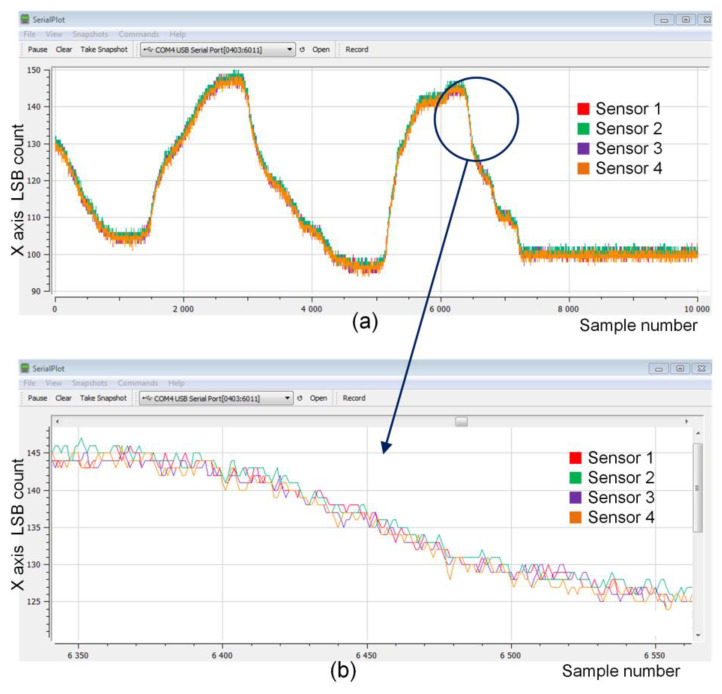
Synchronous data acquisition from four Sensors with accelerometers: (**a**) example of X-axis signals and (**b**) zoomed fragment.

The synchronous data acquisition was tested in a real application. For each of the four Sensors, the ADXL362 accelerometer (see Figure 5a) was connected to the nRF52 SoC via the SPI interface. All the Sensors, placed approximately 0.3 m apart, were attached to one common bar that was rotated. Sampling events were triggered by an additional sampling timer implemented in the Sensors at a frequency of 100 Hz (10 samples per SUF). Data samples from each accelerometer (three-axis) collected in the given super frame (SUF_N_) were formed into a packet and sent to the Central during the next super frame (SUF_N+1_). The signals obtained for the X-axis are shown in Figure 18a and the magnified fragment is shown in Figure 18b. The presented waveforms confirm the very good synchronization of the data readings from the Sensors.

### 5.2. Data Transmission Reliability

The reliability of the data transfer is the most significant assessment parameter for any communication network. This is particularly important in saWSNs, where there is no time to retransmit the lost data packets. From Figure 12 and Figure 13, it can be seen that, even for small transmitter output power (TXPWR ≥ −4 dBm), a PER of ≤1% can be obtained, and it does not depend significantly on DR and BN. Moreover, for TXPWR ≥ 0 dBm, the PER was less than about 0.1%. Therefore, the dependence between the packet error rate and the distance between nodes was tested with a constant TXPWR of—12 dBm. As can be seen in Figure 14, even for this low value of transmission power, for a distance of ≤2 m, the PER was less than approximately 2%. In each case (Figure 12, Figure 13 and Figure 14), a decrease in DR resulted in a decrease in the PER. The results confirmed that reliable data transfer without the loss of the radio packet iwa possible at a distance of up to 2 m between the Central and Sensor. These are the distances that usually occur in WBAN networks.

### 5.3. Energy Saving

Another main aim of this study was to save the sensor node energy. Wireless sensors are often battery-powered, and the operating time between recharges or battery replacement is expected to be as long as possible. For this purpose, various hardware and software techniques that minimize the energy consumption have been adapted. In the proposed solution, the ULINKplus adapter was used to optimize the nRF52 SoC code to enhance the power efficiency.

An analysis of the average power consumption of the radio module (Figure 16 and Figure 17) showed that, for small values of BN (approximately 10), the power was slightly dependent on BN, TXPWR, and DR. This was because the operating time of the nRF52 transceiver in the TX mode was comparable to that in the RX mode. Thus, for a small number of BNs, extending the duration of the Super Frame would be beneficial. However, as BN increased (above 10), TXPWR and DR had an increasing influence on the energy consumption. This dependence was less visible at DR = 2 Mb/s, because a higher data rate caused a decrease in the transmission time and, thus, a reduction in energy consumption. The results of the average power measurement in the radio module (EYSHJNZXZ) within 8 s are presented in Table 5.

For DR = 2 Mb/s, there was a lower power consumption than that for DR = 1 Mb/s, whereas for TXPWR ≥ 0 dBm, the PER did not depend much on DR (see Figure 12 and Figure 13), and to save energy, it was more beneficial to set a higher DR and TXPWR. For example, for BN = 1000, the energy profit between DR = 1 Mb/s and 2 Mb/s was greater than the TXPWR difference between −4 dBm and 0 dBm (see Table 5). Further energy savings are possible, for example, by manipulating the times that the Sensors receive sync packets (e.g., every other Super Frame). Additionally, the dynamic management of TXPWR can be introduced.

### 5.4. Proposed Protocol

The proprietary protocol presented in this paper is based on TDMA, the sleep/wake-up techniques, and continuous data synchronization. As a result, a low-energy-consumption protocol was achieved. Periodic resynchronization was established by the sync packet transmission at the beginning of each Super Frame. Very-high-accuracy sampling synchronization (0.8 µs) was possible due to the use of tasks and events programming, as well as a priority interrupt management in the nRF52 SoC. Thanks to the proposed SUF organization, a fixed latency of 2× *t_SUF_* was achieved in the data acquisition. Increasing the *t_SUF_* would increase the latency, but also reduce power consumption. In the proposed SUF configuration, the duration of the SYNC slot was fixed at 3 ms. This was an excessive value, because sending a 12-bytes-length sync packet takes only 224 µs (for DR = 2 Mb/s). Therefore, additional data packets (e.g., with configurations, information, or requests) could be transferred in the SYNC slot from the Host to the Device or vice versa. Also, during the same SYNC slot, it was possible to send a sync packet twice. Thanks to this, the risk of losing the sync packet in Devices was reduced. Unfortunately, the loss of this packet would result in the loss of Device data for the previous SUF frame. But, in some applications, such as WBANs, data synchronization may be more important than temporary data loss. In addition, the proposed protocol could be modified so that the loss of one or a maximum of several sync packets would not stop the process of sampling and sending data from the Device to the Host. During this time, there will be no significant desynchronization of data acquisition, especially for *t_SUF_* < 1 s. Of course, this depends on the stability of the clock used and the sampling frequency. 

Another feature of the proposed protocol is the ease of configuring the number of devices. It can vary from one to several dozen and depends on the required throughput for the data of an individual Device and the duration of *t_SUF_*. However, there is one limitation: *t_SUF_* should be an integer multiple of *t_SAMPDEVN_* (see Figure 3). Removing this limitation is possible, but would require the other synchronization method of the sampling timer.

The advantage of the protocol is the possibility to maintain energy-efficient and high-throughput communication with very fast sensor node responses (in time ≤ *t_SUF_*) in the case of a temporary loss of communication.

Although the proposed protocol is based on the very well-known TDMA technique, a novel scheme for its use was proposed. First of all, compared to other solutions based on TDMA, the proposal does not require sending any synchronizing information (e.g., timestamps or coordinates) or performing any calculations. It does not focus on time synchronization or clock correction, but only on the synchronization of sampling moments. The moment of sync packet reception is important, not its content. In connection with the hardware (not polling) triggering processes (e.g., triggering the sampling timer) in a SoC, an accuracy of data acquisition synchronization below 1 µs can be achieved, which is not possible in many traditional protocols. In this approach, the CPU activity time is minimized, and, at the same time, a significant amount of energy is saved.

Furthermore, the proposed solution does not require any additional hardware and ensures a low computational cost. The implementation consists of only a radio transceiver and a microcontroller with nested interrupts support. The software implemented in the microcontroller does not require complex calculations. It is mainly based on one-time configuration and events handling. This is a light-weight protocol based on TDMA with a very high precision of sampling events synchronization and a low energy consumption.

It is worth emphasizing that, in this solution, the operation of individual nodes, including their loss, does not affect the operation of the entire WSN. The node can be turned off and go into the energy-saving mode at any time. It can also be included in the network organization immediately (after one SUF frame). Thanks to this, nodes can dynamically disconnect/connect, for example, based on motion detection, the threshold values of measured signals, etc. This offers even additional energy-saving opportunities.

Another feature that distinguishes this solution from other protocols is the possibility of real-time data transmission. During the SUF frame duration, the Sensor collects the samples of the measured signals in a buffer and sends them in the next SUF frame. As a result, the Central receives those samples in real time with a constant delay equal to 2 × *t_SUF_*, which is the same for all nodes.

Summarizing the discussion on the proposed protocol, it is worth noting that, despite the fact that there are many traditional protocols (e.g., Bluetooth Low Energy, ZigBee, 802.15.4), the TDMA technique is still popular. In [60], a TDMA approach was applied to molecular communication via diffusion-based nano-sensor networks for data-gathering applications in in-body medical systems. The design of a hybrid LPWAN mesh network using a custom TDMA for IoT applications that delivers data over the distance of several kilometers with only low-power nodes was presented in [61]. In [62], a heuristic TDMA scheduling algorithm was presented to achieve optimized time slot scheduling with multichannel communication. A new efficient TDMA scheduling algorithm based on a genetic algorithm was presented in [63]. There are also known new approaches combining TDMA and CSMA (carrier sense multiple access) techniques [55,64].

### 5.5. Technical Analysis of the Proposed Scheme

The proposed protocol can be implemented in a network with different numbers of sensor nodes (devices) and variable SUF durations, however, it has an impact on the performance of the saWSN. As the proposed scheme is dedicated mainly to synchronous data acquisition, an important factor is also the stability of the clock, which determines the moments of the signal sampling.

**Figure 19:**
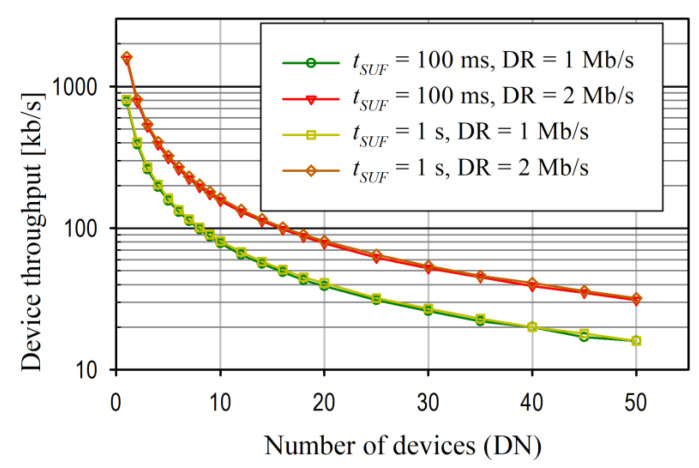
Analysis of maximum device throughput versus number of devices for different *t_SUF_* and DR.

Increasing the number of devices (DN) reduces the duration of the *t_DEV_* slot and, in turn, directly affects the maximum throughput data (MDT) for each device. This relationship is shown in Figure 19. 

The MDT decreased with an increase in the DN and the duration of *t_SUF_*. However, this depended on the DR, which is obvious. For DN = 1, the MDT was close to the DR. For the nRF52 SoC used in the test bench, the on-air maximum packet length was 258 bytes, including a maximum of 255 bytes of payload data. Therefore, the maximum theoretical use of the DR for data transfer was 95%. In real applications, however, the effective throughput is even lower due to the specific protocol implementation (for example, additional time is needed for control, process switching, etc.). In this implementation, the use of DR for effective data transfer was approximately 80% (i.e., DN = 1, *t_SUF_* = 1 s, DR = 2 Mb/s, MDT = 1.58 Mb/s).

In the analysis shown in Figure 19, it is assumed that the durations of all *t_DEV_* slots are the same. However, *t_DEV_* does not have to be uniform for all devices and can be adapted to a specific application (e.g., the max. throughput for Device 1 may be greater than that of Device 2). In all these cases, however, the condition described by Equation (1) must be met.

(1) $$t_{DEV1}+t_{DEV2}{+\ldots +t}_{DEVN}\leq \;{t_{SUF}-t}_{SYNC}-t_{BREAK}$$

**Figure 20:**
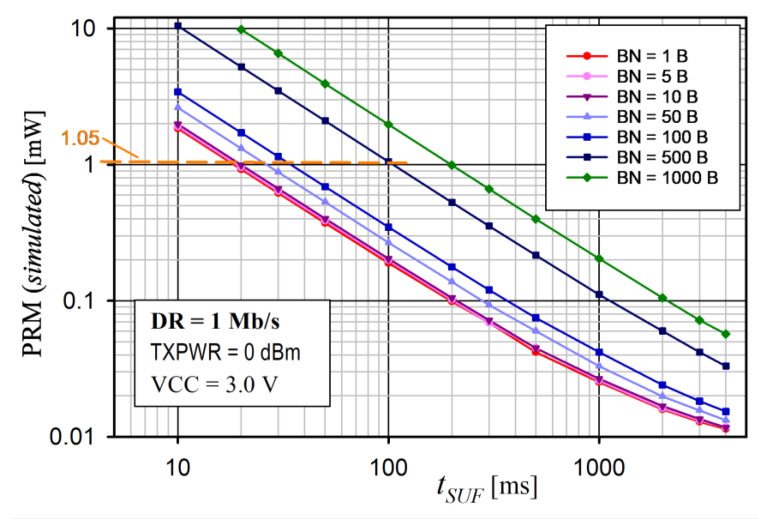
Analysis of average nRF52 SoC power consumption (PRM) versus *t_SUF_* for different sizes of data payloads (BN) and DR = 1 Mb/s.

**Figure 21:**
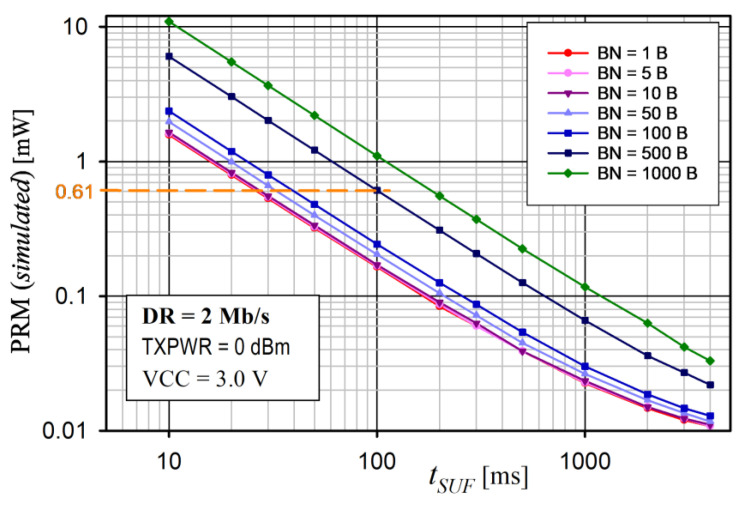
Analysis of average nRF52 SoC power consumption (PRM) versus *t_SUF_* for different sizes of data payloads (BN) and DR = 2 Mb/s.

Another important factor affecting the performance of the proposed saWSN scheme is *t_SUF_*. The *t_SUF_* has an impact on the energy consumption and the accuracy of the data acquisition synchronization. A calculator [65] was used to analyze the influence of *t_SUF_* on the total average nRF52 SoC current for different values of BN and DR. The simulation assumed: VCC = 3.0 V, DC/DC = on, TXPWR = 0 dBm, data packet length extension = on, and length of the sync packet = 12 bytes. The results for DR = 1 Mb/s and 2 Mb/s are shown in Figure 20 and Figure 21, respectively.

In all cases, the power consumption decreased with increasing *t_SUF_*. This was due to the fact that, as *t_SUF_* increased, sync packets were transmitted less frequently, so the device remained in the sleep mode for longer. This is particularly important for small amounts of data transferred (i.e., low BN). Therefore, to save energy, *t_SUF_* should be longer for small values of BN. Increasing DR also reduced PRM, as the transmission time was shorter. For example, for *t_SUF_* = 100 ms and BN = 500 B, the PRM was 1.05 mW and 0.61 mW for DR = 1 Mb/s and 2 Mb/s, respectively.

Although increasing *t_SUF_* saves energy, at the same time, the data latency increases. Moreover, an increase in *t_SUF_* adversely affects the synchronization of data acquisiton. Because data synchronization is very important in this research, the impact of the sampling frequency (*f_S_*), *t_SUF_*, and clock stability on the synchronization error was analyzed. For this purpose, the maximum sampling period error (*SPE_max_*) that could occur in the device was determined. According to Figure 3, it was assumed that the first sampling moments in all devices were synchronized with each other with an accuracy (*s_A_*) of about 0.8 µs (see Section 5.1). It was also assumed that, in each device, an extra timer with a nominal frequency *f_LFXO_* = 40.000 kHz was used for the sampling process. The *SPE_max_* is described by Equation (2).

(2) $${SPE}_{MAX}=\left(t_{SUF}{\;\times \;f}_{S}-1\right)\times f_{LFXO}\times d+s_{A}\times f_{S}$$

where:

*S_A_*—synchronization accuracy

$$d=\frac{1}{\left(f_{LFXO}-\frac{f_{LFXO}\;\times \;v}{10^{6}}\right)}-\frac{1}{\left(f_{LFXO}+\frac{f_{LFXO}\;\times \;v}{10^{6}}\right)}\;$$

*v*—*f_LFXO_* frequency variation in ppm

**Figure 22:**
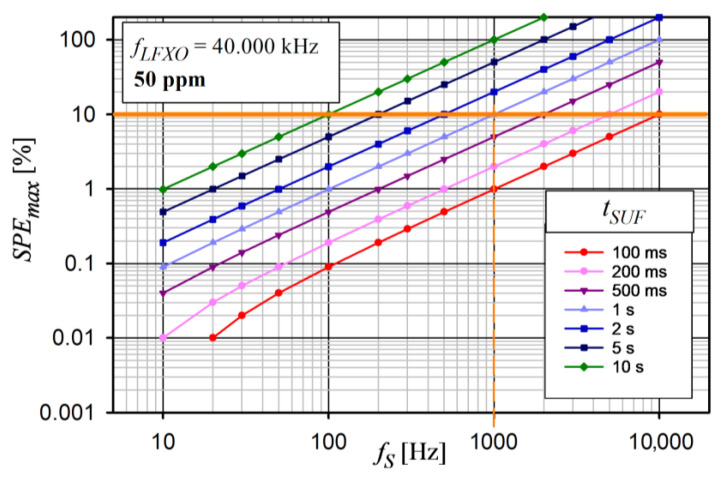
Analysis of maximum sampling period error versus *f_S_* for different *t_SUF_* and constant *f_LFXO_* stability of 50 ppm.

**Figure 23:**
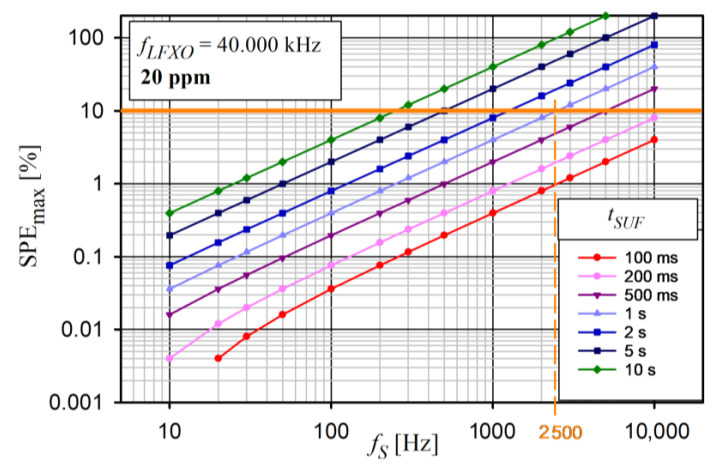
Analysis of maximum sampling period error versus *f_S_* for different *t_SUF_* and constant *f_LFXO_* stability of 20 ppm.

The *SPE_max_* analysis for *f_LFXO_* clock stabilities of 50 ppm and 20 ppm is shown in Figure 22 and Figure 23, respectively.

An increase in *f_S_* caused an almost linear increase in the *SPE_max_* error in each case. Moreover, *SPE_max_* grew very quickly with the increasing duration of SUF (*t_SUF_*). Therefore, to obtain the expected accuracy of the data acquisition synchronization for the selected *f_S_*, the *t_SUF_* should be carefully selected. The *SPE_max_* was directly influenced by the clock stability. For example, for *SPE_max_* not to exceed 10% for *t_SUF_* = 1 s, the maximum *f_S_* is 1 kHz and 2.5 kHz for the clock stabilities of 50 ppm and 20 ppm, respectively. The value of *SPE_max_* ≥ 100% means that, in the saWSN, there is a desynchronization of the sampling moments by at least one sampling period, so it is a case that should not occur.

In the proposed type of sensor node, it is worth paying attention to one factor that affects the energy consumption. It is a digital interface for communication between the SoC and another on-board sensor (e.g., digital accelerometer). Typically, a three- or four-wire SPI or two-wire I2C interface is used. Although an I2C is a user-friendly interface, it consumes more energy than an SPI. This is because the I2C operates at a much lower clock frequency than the SPI (typically 400 kHz versus 10 MHz), requiring longer CPU or DMA controller activity. Moreover, the I2C bus requires pull-up resistors (*R_p_*) (typically 2 kΩ for 400 kHz). A low state on the I2C bus forces a quite high current to flow through these *R_p_*, for example, for V_I2CBUS_ = 3 V, 2 × 2 kΩ, it is as high as 3 mA. This value is quite large compared to the nRF52 SoC radio (7.1 mA for TXPWR at 0 dBm [58]). Therefore, to save energy in the sensor node, it is better to use the SPI interface than I2C.

The energy consumption of the sensor node may also be influenced by other factors connected with the SoC type. For example, in the used nRF52 SoC, the Real-Time Counter (RTC) tick event should be disabled, because it increases the energy consumption, even if the RTC interrupt is turned off. This is particularly important for the energy-saving mode of operation (i.e., sleeping mode).

The above-mentioned details, the use of hardware features such as PPI, DMA, and other autonomous controllers, the sleep and fast wake-up technique, tasks and events programming, proper interrupts usage, including nested ones, avoiding the polling technique, and optimizing the code to minimize the energy consumption using special debuggers (with power measurement features) are design elements that may be defined as deep energy-efficient coding (DEEC). DEEC allows for the design of various types of sensors for energy-aware applications.

### 5.6. Complexity and Computational Performance of the Proposed Scheme

The proposed type of sensor node can be used as an IoT node with strict constraints in terms of memory, power, computational capability, and communication resources. The implemented protocol is light-weight and requires a low computing power. The implementation of the proposed protocol in the nRF52 SoC and Keil MDK environment requires only 8.6 kB and 1.1 kB of program and data memory, respectively. Moreover, due to the use of the PPI technique, DMA, and a Cortex-M4 nested vectored interrupt controller, the load on the nRF52 CPU is very small. These practices save energy and allow the other algorithms to run in parallel. However, the use of PPI and DMA techniques is not necessary. All operations can be implemented in software. Unfortunately, the exclusive use of software solutions worsens the accuracy of synchronization and increases the CPU load and energy consumption.

**Figure 24:**
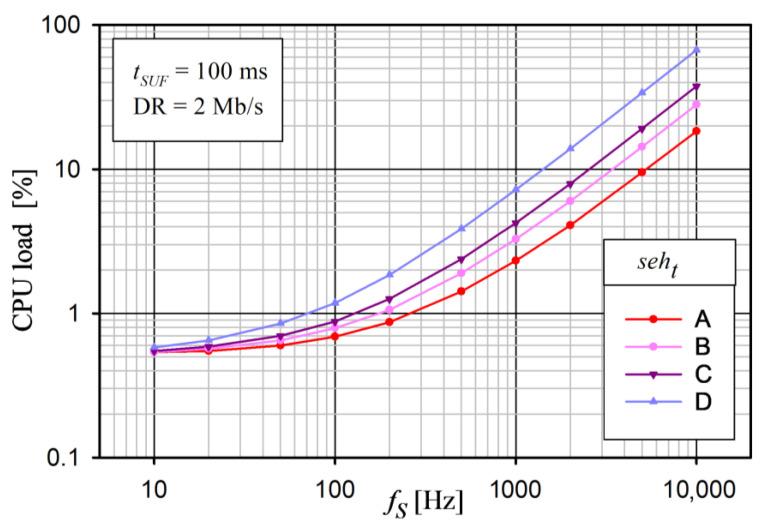
Analysis of CPU load versus *f_S_* for various sampling event handler time (*seh_t_*) (A = 128 clock ticks (c.t.) = 2 µs, B = 640 c.t. = 10 µs, C = 1280 c.t. = 20 µs, D = 3200 c.t. = 50 µs).

In order to determine the computational complexity of the proposed scheme, the CPU execution cycles (clock ticks) were measured. For this purpose, a Data Watchpoint and Trace (DWT) cycle counter located at the core of the Cortex-M4 processor was used. First, the clock ticks (*n_ct_*) and execution times (*e_t_*) for the initialization procedures (performed once at start-up) were measured. For the proposed solution, they were very small, i.e., *n_ct_* = 5204 and *e_t_* = 81 µs (for CPU clock = 64 MHz). However, the number of clock ticks required to perform all procedures during an elementary SUF frame is more important. For this purpose, *n_ct_* and *e_t_* were measured in the sensor nodes between successive sync packet reception events (marked with a dashed red line in Figure 3) for *t_SUF_* = 100 ms and DR = 2 Mb/s. The measurements were carried out for different sampling frequencies and different sampling event handler times (*seh_t_*), with the assumption that two bytes of data were sent for one sample. For each case, 30 measurements were made, and the mean value was calculated. The results for *seh_t_* = 2 µs are presented in Table 6 and Figure 24.

The CPU activity was calculated as the ratio of *n_ct_* with the sleep mode to *n_ct_* without the sleep mode approach. If the CPU is always active, approximately 6.5 million clock ticks are required during one SUF frame. However, it should be noted that, in this case, the processor is active for many periods in the SUF, during which it does not perform any tasks. Therefore, to estimate the real computational complexity of the proposed solution, the processor should be put into sleep mode if it does not perform any tasks.

**Table 6:** Selected results of computational complexity ^1^.

Sampling Events Per One SUF	*f_S_* [Hz]	*n_ct_*	*e_t_* [ms]	CPU Activity [%]
0 ^2^	-	33,067 ^2^	0.52 ^2^	0.51 ^2^
1	10	6,473,031 ^3^	101.14 ^3^	100.00 ^3^
1	10	34,702	0.54	0.54
10	100	44,941	0.70	0.69
100	1000	150,062	2.34	2.32
1000	10,000	1,186,153	18.53	18.34

^1^ Results for *t_SUF_* = 100 ms, DR = 2 Mb/s, CPU clock = 64 MHz, *seh_t_* = 2 µs. ^2^ Sampling process disabled (no sampling events). ^3^ CPU of the nRF52 always in the active mode (without the sleep mode approach).

As shown in Table 6, only approximately 33 thousand clock ticks are needed for the protocol handling algorithm and only approximately 45 thousand clock ticks are required to gather data at the frequency of 100 Hz (at *seh_t_* = 2 µs).

The CPU activity measured with the sleep-mode-enabled approach can be taken as the average CPU load for a given microprocessor and its operating frequency (in this case—nRF52, 64 MHz). The CPU load for the proposed solution versus the sampling frequency for various *seh_t_* is shown in Figure 24.

The *seh_t_* is the time needed to handle a single event of the sampling process, for example, variant A is the time needed to read the Analog-to-Digital Converter (ADC) and trigger it again in the nRF52 SoC. Other variants (B, C, and D) were simulated. It can be seen that, at a low *f_S_* (<200 Hz), the CPU load is very small (<2%) and depends little on *f_S_* and *seh_t_*. In these cases, the CPU load is mainly due to the protocol handling. However, for a higher *f_S_*, the CPU load increases and depends on the value of *seh_t_*.

The evaluation of the computational complexity showed that even a simple low-cost microcontroller can be used for the proposed scheme. The use of a high-performance microcontroller (such as Cortex-M4) in the scheme allows for the parallel running of complex algorithms, for example, for digital signal processing. The two basic elements required to implement the proposed solution are a microcontroller and a radio transceiver. In the past, these were usually separate chips, but today, they are SoCs, such as nRF52 and nRF53 series (Nordic Semiconductor), CC1352 series (Texas Instruments), EFR32BG series (Silicon Labs), BlueNRG series (STMicroelectronics), SmartBond^TM^ series (Dialog Semiconductor), and many others. Besides the many obvious advantages of SoCs (e.g., small dimensions and lower costs), they have many features that are important for energy saving and program code simplification. This results from the fact that the integrated transceiver is a peripheral device of the microcontroller, and its operation is supported by the hardware (e.g., DMA, peripheral interconnect feature).

In terms of the power supply, the proposed sensor node does not have any special requirements. The power supply is typical, similar to that of Bluetooth Low Energy (typically from 1.8 V to 3.6 V), and depends mainly on the SoC used. The maximum peak current mainly depends on the transmitter output power (in this implementation, it is approximately 10 mA for TXPWR = +4 dBm). However, the current of the SoC in sleep mode is important, because the average power consumption largely depends on it.

The proposed scheme also has low communication resource requirements. They only depend on the type of interface needed to communicate with the devices connected to the sensor node (e.g., accelerometer and digital analog front end). The most common are popular interfaces, such as I2C, SPI, and UART.

### 5.7. Synchronization Accuracy and Energy Efficiency Comparison with Related Works

The solution implemented in the proposed sensor node was compared with other related works in which the results of the average power consumption and/or data synchronization accuracy were given. This comparison is presented in Table 7. Unfortunately, not all data can be linked due to insufficient information. In such cases, “n/r” was entered. As shown, data synchronization accuracy at the µs level is possible. In the proposed solution, it was 0.8 µs, which is a very good result.

Because power consumption largely depends on the throughput data, the relationship between them is also presented. Although there is a very large variance in the results, in many cases [22,40,49,66], it is approximately 1÷2 mW × (kb/s)^−1^. In the proposed solution, it is 0.015 and 0.021 mW × (kb/s)^−1^ for transmitter output powers of 0 dBm and +4 dBm, respectively.

### 5.8. Limitations of the Proposed Scheme

The proposed scheme for wireless sensor networks also has the following limitations.

1. All slots in the protocol follow one another in a predefined order, which cannot be changed while the network is running. This can only be changed at the protocol implementation stage.
2. No changes in the protocol are possible when the system is configured. In some cases, for example, when additional information in the synchronization packet is required (and, consequently, the packet length will increase), starting a new implementation of the protocol will be necessary (but only to a small extent regarding the sampling process).
3. No retransmission occurs in the case of packet loss. For this reason, gaps in data streams may occur.
4. The duration of the Super Frame (*t_SUF_*) should be an integer multiple of *t_SAMPDEVN_*.
5. The proposed solution is only suitable for networks with a fixed star configuration.

## 6. Conclusions

In this study, a proprietary protocol for synchronous data acquisition in real-time applications of a WSN was proposed. The analysis of the proposed solution was performed in a real test bench. As a hardware design, a commonly available SoC nRF52 operating in the ISM 2.4 GHz band was used. The experiments confirmed a very high sampling synchronization accuracy (at the level of 0.8 µs) and ultra-low power consumption (only 15 µW per 1 kb/s throughput data and for transmitter output power of 0 dBm). The proposed solution exhibited a good performance in terms of functionality, communication, and energy efficiency because of the sleep/wake-up techniques, tasks and events programming, as well as the DMA and the nRF52 SoC programmable peripheral interconnect feature, minimizing the CPU activity. Additionally, optimization of the sensor node firmware was considered and the ULINKplus adapter was used, which enabled deep energy-efficient coding. This adapter, with energy measurement and simultaneous debugging functions, creates new possibilities for designing energy-efficient and high-performance electronics, especially for battery-powered or battery-free devices.

The proposed solution is designed for systems with periodic data acquisition. When the samples’ data buffer is full, the sensor data are packetized and sent as one frame in a predefined slot of the TDMA cycle. This requires, however, prior planning of the entire system, and no changes can be enacted while the network is running. In the proposed protocol, there is no retransmission due to packet loss that may occur in the radio channel. The radio channel condition is indeed the only factor affecting the transmission reliability. Data collisions do not occur, because TDMA is used. Thus, the complex mechanisms for data anticollision and reliable data transmission like in Bluetooth or the ZigBee solution are not necessary. The proposed solution is dedicated to WSN applications, especially for WBAN, where synchronous data acquisition is more important than the temporary loss of data packets, for example, in a novel cuff-less continuous non-invasive blood-pressure-monitoring method based on pulse arrival time. The effectiveness of the proposed solution was evaluated through experiments conducted under a lab-controlled test bench. The results demonstrated an improved energy efficiency, data synchronization accuracy, and high throughput for real-time data. A comparison with other proposals is presented in Table 7. Such sensors can be used as edge devices in the rapidly developing medical IoT, particularly when there is a need for synchronous and accuracy continuous or periodic data acquisition.

Future work will include data encryption and large-scale testing using physiological signal acquisition. Mechanisms for the retransmission of lost packets and the adjustment of the output power according to the estimated RSSI value will also be considered.

## Figures and Tables

**Table 1:** Results of central and sensor clock base frequency measurements ^1^.

Freq. [mHz]	Central A ^2^	Central B ^3^	Sensor 1	Sensor 2	Sensor 3	Sensor 4
Mean	−9	+404	+216	+423	+573	+460
Min	−120	+402	+216	+421	+569	+459
Max	+180	+406	+217	+425	+576	+461
StdDev	110.04	1.44	0.29	1.33	2.18	0.78

^1^ The results (except StdDev) are shown as a difference to a frequency of 20 kHz (measured with an accuracy of 1 mHz). ^2^ Based on nRF52-DK. ^3^ Based on Sensor board.

**Table 2:** Results of Central and Sensor SUF durations (*t_SUF_*) ^1^.

Time Interval	Central	Sensor 1	Sensor 2	Sensor 3	Sensor 4
Mean [ns]	+422	+421	+420	+439	+440
StdDev [ns]	40	75	77	87	86

^1^ Mean is shown as a difference to a time interval of 100 ms (measured with accuracy of 1 ns).

**Table 3:** Results of SUF synchronization achievement (*t_M_*_1_).

Time Interval [µs]	Central–Sensor Distance [m]	Sensor 1	Sensor 2	Sensor 3	Sensor 4
Mean	0.5	264.269	264.515	264.393	264.448
StdDev		0.051	0.046	0.054	0.061
Mean	1.0	264.292	264.524	264.380	264.462
StdDev		0.053	0.049	0.054	0.074

**Table 4:** Results of sampling synchronization achievement (*t_M_*_2_).

Time Interval [µs]	Sensor 1	Sensor 2	Sensor 3	Sensor 4
Mean	311.271	312.097	311.828	311.717
StdDev	0.096	0.087	0.079	0.092

**Table 5:** Selected results of average current measurement in radio module within 8 s (*t_SUF_* = 100 ms, VCC = 3.0 V, DC/DC on).

Payload Size (BN) [bytes]	Throughput [kb/s]	Max *f_S_* ^1^ [Hz]	TXPWR [dBm]	IRM [µA] @ DR = 1 Mb/s	IRM [µA] @ DR = 2 Mb/s	Power [mW] @ 1 Mb/s	Power [mW] @ 2 Mb/s
10	0.78	66.7	−4	73	69	0.22	0.21
10	0.78	66.7	0	77	70	0.23	0.21
10	0.78	66.7	+4	84	74	0.25	0.22
100	7.81	666.7	−4	114	89	0.34	0.27
100	7.81	666.7	0	126	95	0.38	0.29
100	7.81	666.7	+4	154	109	0.46	0.33
1000	78.13	6666.7	−4	582	340	1.75	1.02
1000	78.13	6666.7	0	691	393	2.07	1.18
1000	78.13	6666.7	+4	948	526	2.84	1.58

^1^ Frequency sampling assuming that one sample = 12 bits (typical resolution of an analog-to-digital converter built into the microcontroller.

**Table 7:** Comparison of the proposed solution with other related works.

Reference Work	Radio Band	RF Transceiver	Protocol	Super Frame Duration	Synchronization Accuracy	Power Consumption @ Throughput Data	Power Consumption [µW]/throughput Data [kb/s]
Marinkovic et al., 2009, [49]	433 MHz	ADF7020	Proprietary	1 s	n/r	2.04 mW @ 1250 b/s	1671
Mo et al., 2012, [22]	2.4 GHz	n/r	ZigBee	n/a	<24 ms	19.98 mW @ 15840 b/s	1292
Mo et al., 2013, [66]	2.4 GHz	n/r	Proprietary	n/r	6 ms	22.2 mW @ 14400 b/s	1579
Vanveerdeghem et al., 2014, [67]	2.4 GHz	ADF7242	Proprietary	n/r	n/r	90 mW @ 25 packet/s	n/r
Bernhard et al., 2015, [48]	2.4 GHz	nRF51822	Proprietary	0.1 s	7.6 µs	n/r @ 320 b/s	n/r
Morrison et al., 2015, [52]	915 MHz	nRF905	Proprietary	n/a	n/a	0.19 mW @ 3.2 b/s	60,800
Milenković et al., 2016, [68]	2.4 GHz	CC2420	ZigBee	1 s	30.5 µs	8.31 mW @ 1920 b/s	4432
Pflugradt et al., 2016, [40]	2.4 GHz	CC2560	Bluetooth	n/a	30 µs	100 mW @ 96000 b/s	1067
Chongqing Zhang et al., 2016, [69]	2.4 GHz	CC2520	Proprietary	n/r	n/r	2.6 mW @160 b/s	16,640
Wang et al., 2017, [44]	2.4 GHz	CC3200	WiFi	n/a	n/r	847.3 mW @ 1 kHz	n/r
Shang Gao et al., 2019, [47]	Sub-1-GHz	SX1278	LoRa	n/a	5 µs	n/r @ n/r	n/r
Lewandowski et al., 2019, [70]	2.4 GHz	n/r	ZigBee	0.5 s	n/r	9.14 mW @ n/r	n/r
Asgarian et al., 2022, [71]	2.4 GHz	nRF51822 or nRF52832	BLE ^1^	n/a	0.3 µs ^2^	n/r @ n/r	n/r
Zong et al., 2022, [72]	n/r	n/r	Proprietary	1 s	1 µs	n/r @ n/r	n/r
Cerone et al., 2022, [73]	868 MHz	CC1310	Proprietary ^3^	n/a	<500 µs	21.45 mW @ n/r	n/r
This work	2.4 GHz	nRF52832	Proprietary	0.1 s	0.8 µs	1.18 mW @ 78,130 b/s, TXPWR = 0 dBm 1.58 mW @ 78,130 b/s, TXPWR = +4 dBm	15 21

n/a—not applicable; n/r—not reported. ^1^ Modified Bluetooth Low Energy (BLE). ^2^ Per 60 s. ^3^ Additional synchronization module is required.

## Data Availability

Data are contained within the article.
